# Isosteviol Sodium Promotes Neurological Function Recovery in a Model of Spinal Cord Injury in Rats

**DOI:** 10.1002/iid3.70110

**Published:** 2025-01-09

**Authors:** Tongxia Zhang, Tao Zhang, Han Yu, Lingyi Chi

**Affiliations:** ^1^ Research Institute of Neuromuscular and Neurodegenerative Diseases and Department of Neurology Qilu Hospital of Shandong University Jinan Shandong People's Republic of China; ^2^ School of Basic Medical Sciences Cheeloo College of Medicine Shandong University Jinan Shandong People's Republic of China; ^3^ Shandong Qidu Pharmaceutical Co. Ltd. and Shandong Provincial Key Laboratory of Neuroprotective Drugs Zibo Shandong People's Republic of China; ^4^ Department of Neurosurgery Qilu Hospital of Shandong University and Institute of Brain and Brain‐Inspired Science Shandong University Jinan Shandong People's Republic of China; ^5^ Shandong Key Laboratory of Brain Function Remodeling Jinan Shandong People's Republic of China

**Keywords:** apoptosis, inflammation, isosteviol sodium, oxidative stress, spinal cord injury

## Abstract

**Background:**

Traumatic spinal cord injury (SCI) is an incurable condition that is the largest cause of disability. In previous studies, Isosteviol sodium (STVNa) has been shown to protect rats against acute focal cerebral ischemia; however, the effects of STVNa on SCI recovery in rats remain unknown.

**Methods:**

STVNa was given intraperitoneally after SCI to see if it had any neuroprotective benefits. On Days 7, 14, 21, and 28 post‐SCI, functional recovery was measured using the Basso, Beattie, and Bresnahan (BBB) scoring system along with the oblique plate test. Following these evaluations, spinal cord tissues were harvested for analysis. All behavioral testing occurred between 8 a.m. and 3 p.m.

**Results:**

We found that STVNa improved spinal cord functional recovery in rats, as evidenced by enhanced BBB locomotor rating scale, angle of inclination, decreased cavity of spinal cord damage, and neuron death in vivo. In addition, STVNa reduced inflammation in rats following SCI, as demonstrated by a reduction in proinflammatory cytokines such as tumor necrosis factor (TNF)‐α, interleukin (IL)‐6, and interleukin (IL)‐1β. STVNa also reduced oxidative damage in SCI rats by lowering ROS while raising SOD levels.

**Conclusion:**

These findings suggest that STVNa protects SCI rats through a variety of pathways. STVNa, in particular, may benefit the recovery of SCI by reducing oxidative stress and inflammation, leading to enhanced locomotor activity in rats with SCI.

## Graphical abstract

STVNa protects SCI rats through a variety of pathways. STVNa can alleviate neurological function and histological changes of the spinal cord after SCI. STVNa may benefit in the recovery of SCI by reducing oxidative stress and inflammation, leading in enhanced locomotor activity in rats with SCI.

## 1 Introduction

Spinal cord injury (SCI) is a severe medical condition resulting in lifelong impairment, such as paralysis, movement loss, sensory loss, or loss of autonomy below the afflicted area [1]. Every year, thousands of individuals are impacted worldwide, with catastrophic consequences for patients' well‐being and financial stability [2, 3]. SCI can be divided into two stages: primary and secondary damage. Compression, bruising, stretching, and kinking are all symptoms of direct mechanical impingement of the spinal cord. The secondary SCI is defined by the continuation of sequential processes in the first stages that involve free radical generation, delayed calcium infusion, immunological and inflammatory system response, and death of the apoptotic cells [4]. The role of inflammation in SCI has gained increasing recognition, as the initial injury activates the immune response, leading to the recruitment of immune cells and the release of proinflammatory cytokines. This hyper‐inflammatory environment can hinder recovery and contribute to chronic pain and secondary complications, such as infections and spasticity [5]. Currently, methylprednisolone is the most widely used drug in the clinical treatment of SCI. Although its effect is rapid and effective, long‐term use has great side effects, and it is not easy to measure the long‐term results [6]. In addition, neurotrophic factors, oxygen‐free radical scavengers, antioxidants, and other drugs are often used to treat SCI. However, these agents often yield unsatisfactory results due to their limited mechanisms of action. For example, neurotrophic factors mainly support neuronal survival but do not effectively address inflammation or oxidative stress, both of which are crucial after an injury. Antioxidants may struggle to cross the blood−brain barrier, reducing their effectiveness in the central nervous system. Moreover, these treatments often provide only temporary benefits, making it challenging to achieve lasting recovery [7]. Thus, there is an urgent necessity to investigate the intricate immunological processes that govern SCI and to identify comprehensive therapeutic approaches that can address multiple facets of the injury.

Research has demonstrated that traditional Chinese medicines and herbal extracts are useful in the treatment of nervous system diseases (NSDs), such as Parkinson's disease [8], Alzheimer's illness [9], and SCI [10]. Stevioside and analog isosteviol were used for hundreds of years as a safe, traditional, noncaloric sweetener in *Stevia rebaudiana* leaf, of which the toxicity and safety have been well examined [11]. Several research studies showed that isosteviol promotes cardioprotection in a model of type 2 diabetes [12, 13]. Isosteviol sodium (STVNa) is a high‐solubility and bioavailability‐enhanced formulation, which has been demonstrated to reduce cerebral ischemia and mitochondrial dysfunction in vitro, causing ischemia‐reperfusion [14]. STVNa has been shown to reduce proinflammatory cytokines like IL‐6 and GM‐CSF, which could help manage inflammation and support recovery [15]. Additionally, its antioxidant properties combat oxidative stress and mitochondrial dysfunction, protecting nerve cells during and after injury [16]. By targeting multiple harmful processes, STVNa may provide a more effective and comprehensive approach to treating SCI compared to traditional single‐action drugs. However, it is still unknown whether STVNa has neuroprotective effects against SCI. This study examined whether STVNa can support the functional recovery of rats following SCI and the processes underlying it. This study aims to investigate the capacity of STVNa to facilitate functional recovery in a rat model of SCI and to elucidate the underlying biological mechanisms involved in this process. Understanding whether STVNa can enhance recovery from SCI is critical, as it could lead to the development of novel therapeutic interventions that leverage its anti‐inflammatory and neuroprotective properties. By addressing this gap in knowledge, our findings may provide valuable insights for enhancing treatment strategies for patients suffering from spinal cord injuries.

## 2 Materials and Methods

### 2.1 Experimental Animals

**Figure 1:**
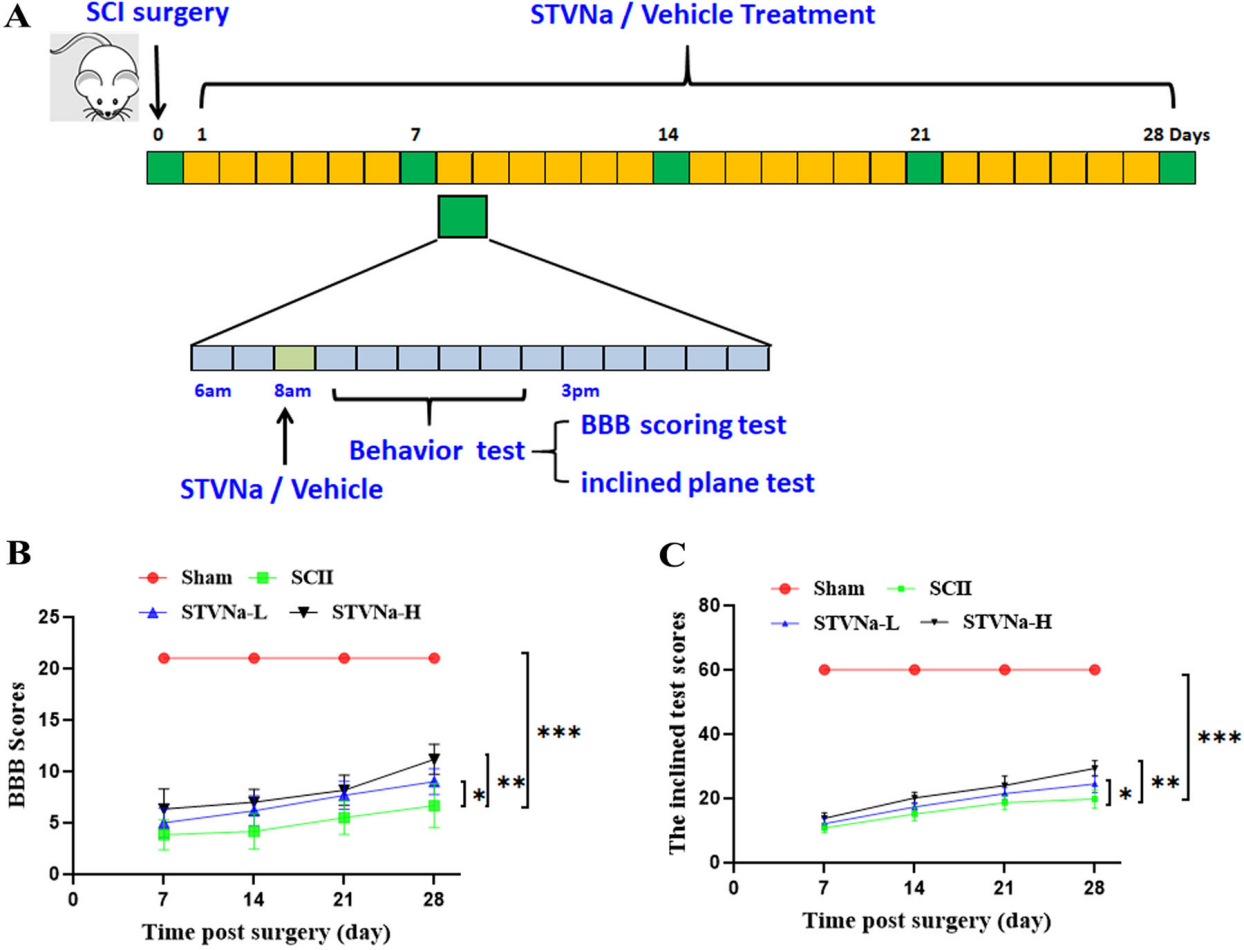
Behavioral recovery of SCI rats increases by STVNa treatment. (A) Experimental design. (B) Basso, Beattie, and Bresnahan (BBB) scores. (C) Inclined plane test scores. The results are mean ± SD (*n* = 6 in each group; *p < 0.05, **p < 0.01, ***p < 0.001).

A total of 24 adult male Sprague−Dawley rats (weight, 200−250 g; age, 9−11 weeks) were randomly assigned into four groups (*n* = 6 per group): Sham, SCI, SCI+STVNa‐L (low dose STVNa, 5 mg/kg/day), and SCI+STVNa‐H (high dose STVNa, 10 mg/kg/day) groups, for dose selection, we refer to some work of others [17, 18]. STVNa was intraperitoneally injected immediately after SCI. The experimental design is summarized in Figure 1A.

### 2.2 STVNa

Isosteviol (Sigma) was dissolved in 70 mL of tetrahydrofuran (THF), and then 8 M NaOH was added. After stirring the mixture for 30 min, it was set aside. The solvent was removed under reduced pressure, yielding 680 mg (2.0 mmol, 95%) of STVNa as a white salt [18].

### 2.3 Rat Model of SCI

The operation was conducted as previously reported [19]. Briefly, rats were anesthetized with 5% isoflurane until unconscious, followed by 3% isoflurane during surgery. An incision was made on the dorsal aspect of the spine from T8 to T10, and the lamina was opened to expose the spinal cord. In the Sham group, only the lamina was opened without spinal shock. The spines were fixed in the rest of the groups, and then the spinal cord was hit (1.5 N) using an IH‐0400 impactor. Following the SCI, the rats were kept alone in a separate cage for a couple of days to monitor their health condition and provide specific postoperative care (antibiotics, analgesics).

### 2.4 Histology

The spinal cord tissue of each group was fixed with 4% paraformaldehyde for 48 h, then embedded in paraffin. The spinal cord was sliced continuously from the center of the injured spinal cord with a thickness of 5 μm. According to the manufacturer's instructions, histopathological examinations were performed using H&E and Nissl staining. Bright‐field images were acquired using light microscopy. The area of SCI and intact neuron counts was quantitatively analyzed by image analysis software (ImageJ).

### 2.5 TUNEL

For TUNEL staining, the paraffin‐embedded sections were dewaxed, rehydrated, and subsequently stained with Colorimetric TUNEL Apoptosis Assay Kit (Beyotime). The slides were incubated with the TUNEL reaction cocktail and streptavidin‐HRP reaction cocktail at 37°C separately for 2 h and 30 min in the dark and counterstained with DAB at 20°C for 30 min. Specimens were observed under a Nikon microscope. The TUNEL‐positivity index was calculated by dividing the number of TUNEL‐positive cells by the total number of cells.

### 2.6 Basso, Beattie, Bresnahan (BBB) Score

The locomotor function of the hind limbs was assessed by BBB score on Days 7, 14, 21, and 28 after spinal injury. Early hind limb joint motion score ranged from 0 to 7 points. The gait and coordination function of the hind limbs were mainly evaluated in the middle and late stages, with a score of 8−13. Finally, the rats' fine movement of the claws was assessed, with the score ranging from 14 to 21 points, and the total normal hind limb motor function was considered 21 points [20]. Following these evaluations, spinal cord tissues were harvested for analysis.

### 2.7 Measurements of IL‐1β, IL‐6, and TNF‐a by ELISA Assays

The inflammatory cytokines IL‐1β, IL‐6, and TNF‐α in the spinal cord tissue of every group were assessed using ELISA according to the instructions provided by the manufacturer.

### 2.8 Determination of Superoxide Dismutase 1 (SOD1), Reactive Oxygen Species (ROS), and Malondialdehyde (MDA) Levels

To detect oxidative stress markers, levels of ROS, MDA, and SOD1 were measured. For ROS, cells or tissues were treated with a fluorescent probe (DCFDA), and fluorescence intensity was assessed using a microplate reader. MDA levels were quantified using the thiobarbituric acid reactive substances (TBARS) assay by measuring absorbance at 532 nm. SOD1 activity was evaluated by monitoring the reduction of nitroblue tetrazolium (NBT) and measuring the decrease in absorbance at 560 nm.

### 2.9 Quantitative Reverse Transcription Polymerase Chain Reaction (RT‐qPCR)

Total RNA was extracted using TRIzol Reagent (Takara, 9108Q) per the manufacturer's instructions. Spectrophotometers at 260 nm measured RNA concentration. PrimeScript RT reagent Kit (Perfect Real Time) (Takara, RR037A) was used to reverse‐transcribe RNA (500 ng) from each sample into cDNA. Finally, an Applied Biosystems 7300 Fast Real‐Time PCR System detected gene expression. The sequences of primers were used as follows: IL‐1β (forward, 5'‐CTTCAAATCTCACAGCAGCATC‐3'; reverse, 5'‐GCTGTCTAATGGGAACATCACA‐3'); IL‐6 (forward: 5'‐ACTTCACAGAGGATACCAC‐3'; reverse: 5'‐GCATCATCGCTGTTCATAC‐3'); TNF‐α (forward: 5'‐ATGGGCTCCCTCTCATCAGTTCC‐3', reverse: 5'‐GCTCCTCCGCTTGGTGGTTTG‐3'). The $2^{-{\mathrm{\Delta \Delta C}}_{t}}$ method was used for quantification. Meanwhile, GAPDH was used as a housekeeping gene.

### 2.10 Western Blot Assay

Protein concentrations were measured using the BCA protein quantification kit (BC3711, Solarbio), and 40 μg protein extracts were subjected to SDS‐PAGE, transferred and sealed, apoptotic markers: Bax (Proteintech, 50599‐2‐Ig, 1:2000), Bcl‐2 (Proteintech, 26593‐1‐AP, 1:1000), cleaved caspase‐3 (Proteintech, 25546‐1‐AP, 1:500); oxidative stress markers: iNOS (Proteintech, 22226‐1‐AP, 1:200), eNOS (Proteintech, 27120‐1‐AP, 1:500), SOD1 (Proteintech, 10269‐1‐AP, 1:5000), NOX2 (Proteintech, 19013‐1‐AP, 1:2000), and β‐actin antibody (Abcam, ab8227, 1:5000) were added, incubated for 24 h at 4°C, then the membrane was washed by TBST, secondary antibodies (anti‐mouse IgG, HRP, 1:5000, ab6728, or anti‐rabbit IgG, HRP, 1:5000, ab6721, Abcam) for 2 h at room temperature, and TBST washed the membrane. The immunoreactive areas were visualized using enhanced chemiluminescence (ECL). Relative optical densities were quantified by ImageJ.

### 2.11 Statistical Analysis

Statistics were analyzed using SPSS22.0. Data were presented as mean ± SD. A nonparametric one‐way ANOVA test with Tukey's posttest was used for statistical analysis. Statistical significance was indicated by *p < 0.05, **p < 0.01, and ***p < 0.001.

## 3 Results

### 3.1 STVNa Improved the Locomotor Function of SCI Rats

It is considered that the locomotor function is a good predictor of spinal cord damage. The effect of STVNa on the rehabilitation of locomotive function was assessed in this study. The experimental design is summarized in Figure 1A. Administering STVNa raised the BBB score, particularly at high doses, while the sham group had a much lower BBB score (Figure 1B). Moreover, the oblique plate test results showed that the STVNa‐H group scored higher than the STVNa‐L group and SCI groups (Figure 1C), showing that the administration of STVNa substantially enhanced the recovery of locomotive function in rats with SCI contusive.

### 3.2 STVNa Attenuates Structural Tissue Damage and Neuron Loss of SCI Rats

**Figure 2:**
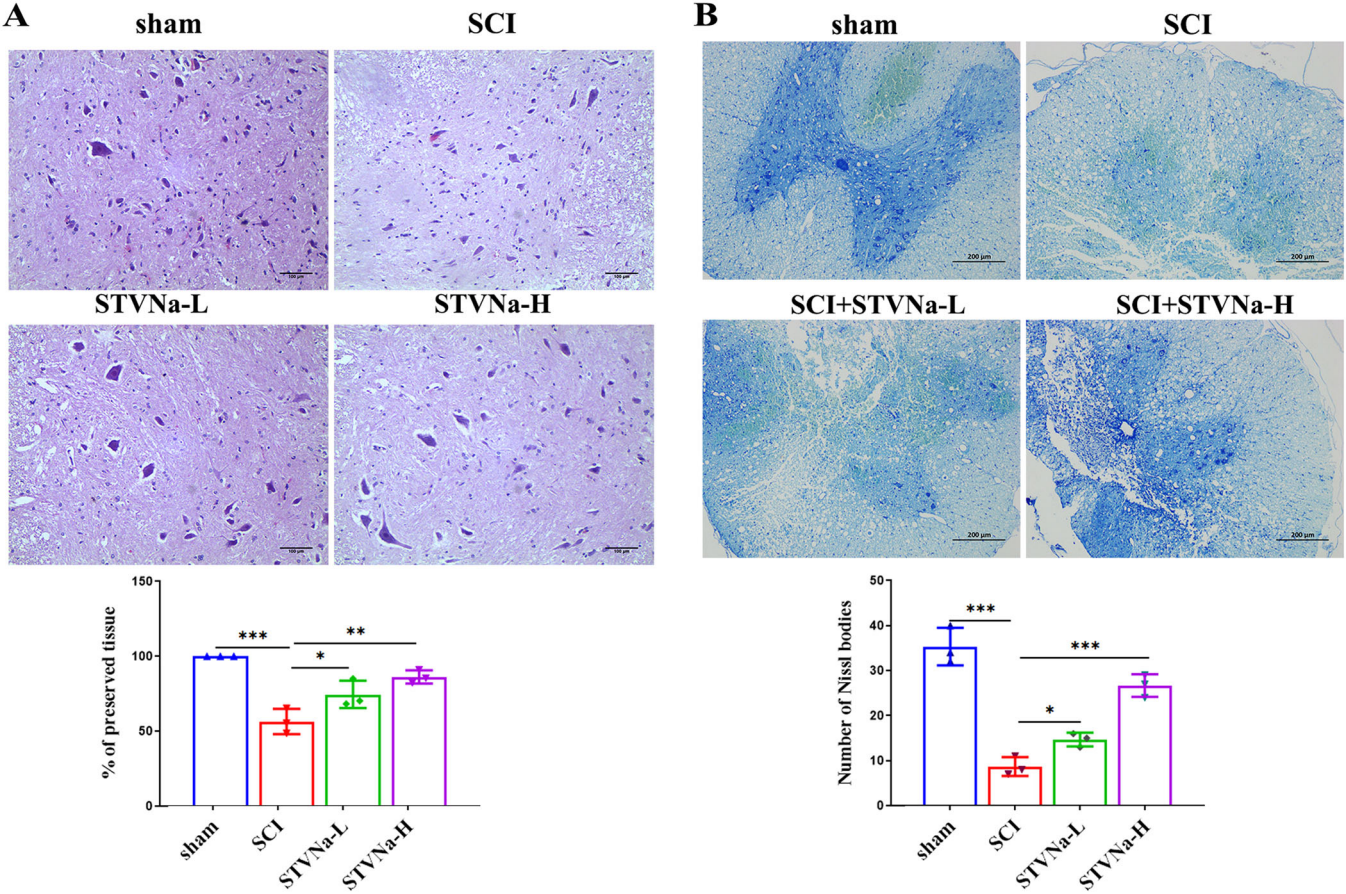
Isosteviol sodium attenuates structural tissue damage and neuron loss in SCI rats. (A) Representative H&E staining micrographs show cavity formation in the sham, SCI, STVNa‐L, and STVNa‐H groups (scale bar = 100 μm). (B) Nissl staining of the spinal tissue sections was collected on Day 28 in sham, SCI, STVNa‐L, and STVNa‐H groups (scale bar = 200 μm). The results are mean ± SD (*n* = 3 in each group; *p < 0.05, **p < 0.01, ***p < 0.001).

We examined the histological morphological alterations in HE and Nissl in each group. As demonstrated in Figure 2A, the rats in the SCI group had seriously damaged central gray material and dorsal white matter in contrast to the sham group. Compared with the SCI group, a diluted central and dorsal white matter cavitation was presented in the treatment group STVNa, showing that STVNa had therapeutic effects on SCI. The number of Nissl bodies has been counted. The STVNa treatment group showed considerable improvements in Nissl body loss and neuronal activity compared to the model group (Figure 2B).

### 3.3 STVNa Inhibited Apoptosis in the Spinal Cord of SCI Rats

**Figure 3:**
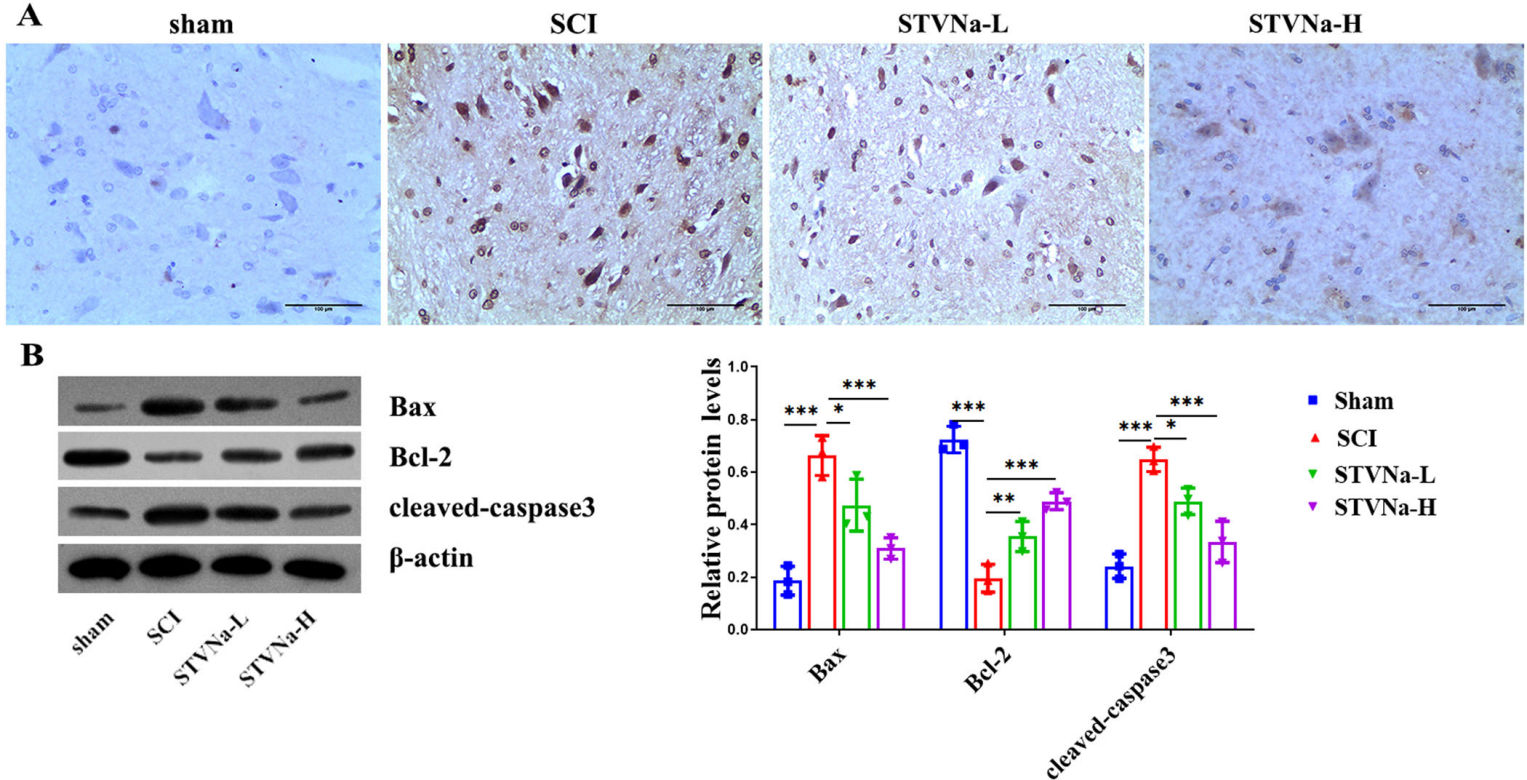
Isosteviol sodium inhibited apoptosis in the spinal cord of SCI rats. (A) Apoptosis cells in the spinal cord of rats were detected by TUNEL staining (magnification at ×200). (B) Western blot analysis of cleaved caspase 3, Bcl‐2, and Bax expression in each group of rats (*n* = 3 in each group; *p < 0.05, **p < 0.01, ***p < 0.001).

Figure 3A shows that the number of apoptotic cells was much lower in the groups treated with STVNa compared to the SCI group, and that this decline was further decreased as the concentration increased. To determine whether STVNa prevents cell death in the brain, we used WB to check for Bcl‐2, Bax, and caspase 3 levels (Figure 3B). These studies show that STVNa attenuates apoptosis neurons.

### 3.4 Effect of STVNa on Inflammation in Rats With SCI

**Figure 4:**
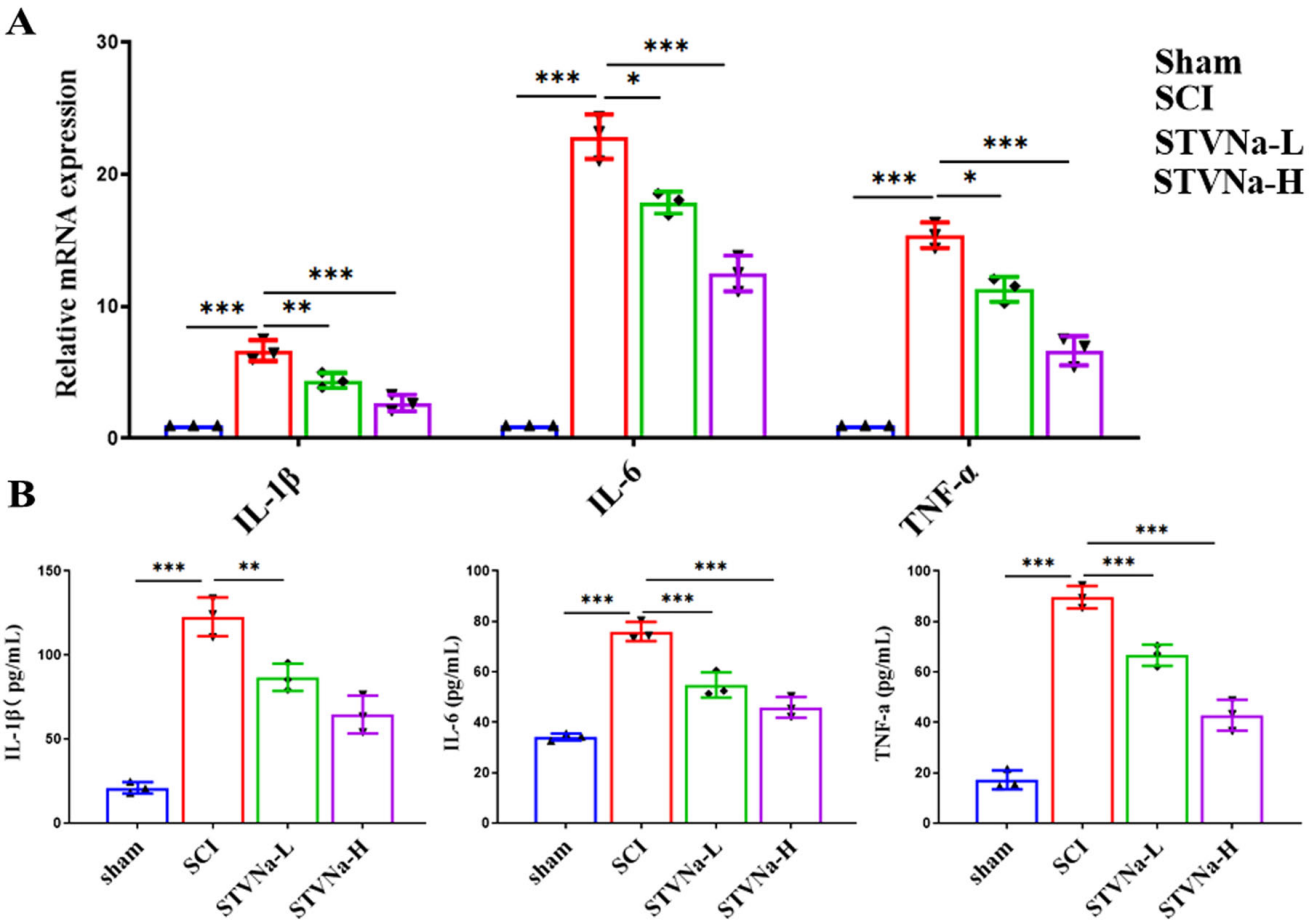
Isosteviol sodium modulates the neuroinflammatory response in rats with SCI. (A) RT‐qPCR detected RNA expression of IL‐1β, IL‐6, and TNF‐α. (B) ELISA assay showed the expression of IL‐1β, IL‐6, and TNF‐α proteins in different groups (*n* = 3 in each group; *p < 0.05, **p < 0.01, ***p < 0.001).

Figure 4A shows that the mRNA expression levels of proinflammatory mediators, including IL‐1β, IL‐6, and TNF‐α, were elevated in the SCI group compared to the sham group. In the STVNa‐treated group, their expression levels were reduced compared to the SCI group (Figure 4A). Furthermore, ELISA results are consistent with qRT‐PCR results (Figure 4B). These findings suggested that STVNa therapy may suppress proinflammatory cytokine response after SCI.

### 3.5 STVNa Suppressed Oxidative Damage in the Spinal Cord of SCI Rats

**Figure 5:**
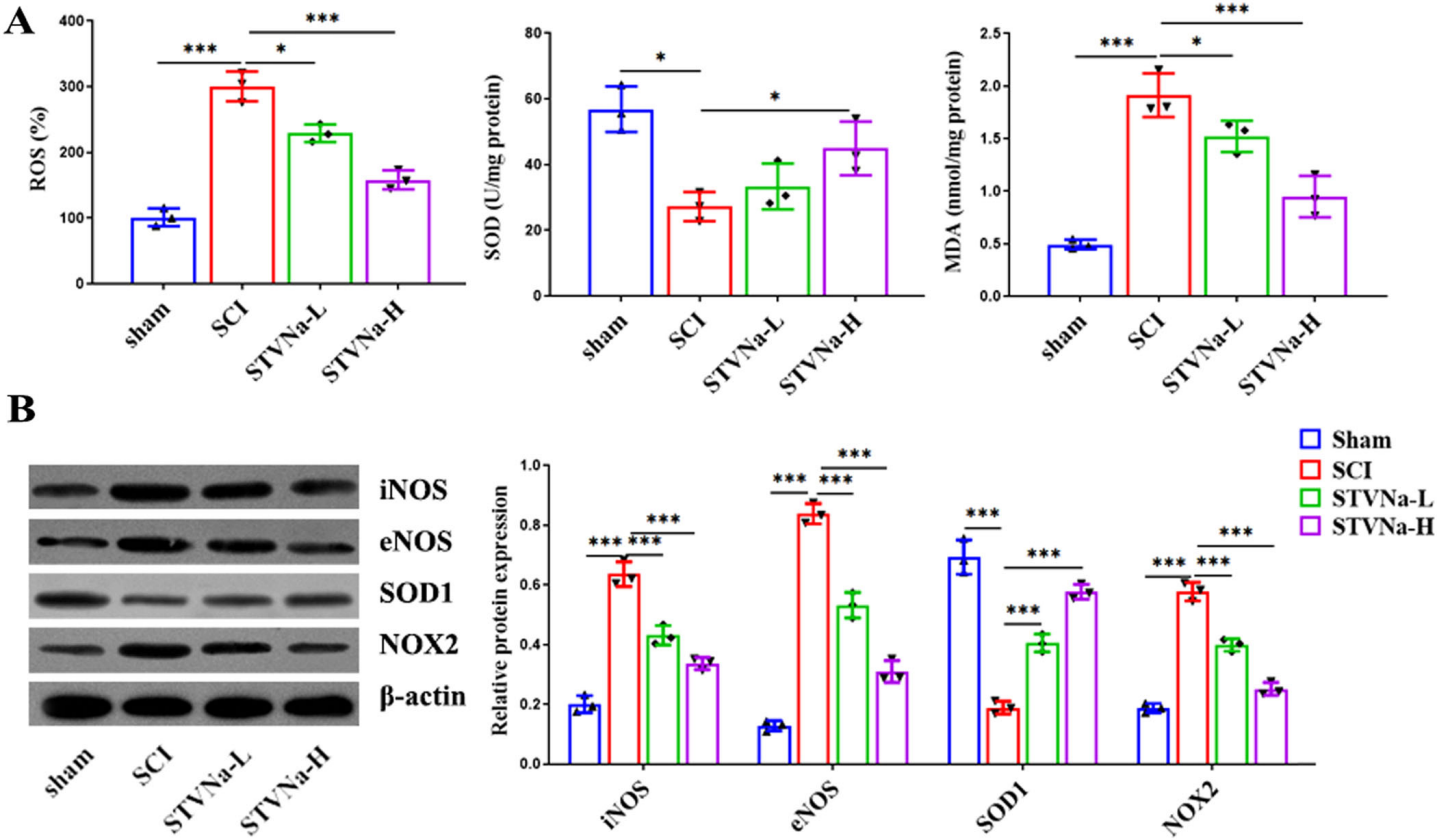
Isosteviol sodium suppressed oxidative damage in the spinal cord of SCI rats. (A) The generation of ROS in different groups. (B) Protein expression of superoxide dismutase (SOD) and (C) malondialdehyde (MDA) in different groups. (D) In each group, western blot analysis was performed to measure iNOS, eNOS, SOD1, and NOX2 protein levels (*n* = 3 in each group; *p < 0.05, **p < 0.01, ***p < 0.001).

We assessed the impact of STVNa on the main oxidizing stress regulators in rats with SCI to clarify probable mechanisms of STVNa‐induced protective benefits against SCI and a decrease in oxidative stress. The results demonstrate that the SCI caused a significant increase in ROS and MDA compared to the sham group, which was largely reversed by STVNa treatment in a dose‐dependent manner. Furthermore, STVNa treatment rescued SCI‐induced inhibition of SOD (Figure 5A). Moreover, after treatment with STVNa, the protein expression of SOD1 was increased, and the protein expression of iNOS, eNOS, and NOX2 was decreased (Figure 5B).

## 4 Discussion

SCI is a paralytic and extremely debilitating illness with a considerable loss of sensory and motor function [21]. Many researchers have focused on drugs inhibiting the function and pathologic recovery of neuronal death following SCI [22, 23, 24, 25, 26]. The objective of this research was to investigate the mechanism behind the effects of STVNa on neurological function after SCI damage in rats. The BBB and inclined plane test findings demonstrated that STVNa therapy considerably enhanced functional motor restoration after damage. In addition, the staining of H&E and Nissl demonstrated that STVNa avoided the loss of motor neurons in the ventral horn. TUNEL staining and western blot assays revealed that following STVNa treatment, the apoptosis rate in the SCI site decreased, accompanied by elevated Bcl‐2 protein levels and reduced expression of Bax and cleaved caspase‐3 proteins. These data suggested that STVNa improves functional recovery after acute SCI by preventing neuron apoptosis.

The prevalence and progression of SCI are connected to oxidative stress [27]. SOD is an antioxidant defense enzyme, whereas MDA, a lipid peroxidation result, indicates oxidative damage. Research shows that STVNa reduces oxidative stress, restores mitochondrial membrane potential (ΔΨm), and maintains calcium homeostasis, protecting against isoprenaline‐induced cardiac hypertrophy [12]. An increase in expression of all three NOS isoforms‐nNOS, iNOS, and eNOS—has been shown in research on spinal cord injuries [28]. In this study, we confirmed that STVNa can significantly alleviate the level of oxidative stress in SCI by detecting levels of oxidative stress markers MDA, SOD, and ROS. The systems responsible for producing and removing free radicals are in a constant state of equilibrium in a healthy body [29]. When this equilibrium is upset, oxidative stress occurs. To stabilize the local microenvironment and forestall more cell damage, STVNa quickens ROS elimination by increasing SOD activity and suppresses MDA. Oxidative stress can activate inflammatory pathways, and conversely, inflammation can promote the generation of ROS, creating a vicious cycle of oxidative stress and inflammation [30]. The inflammatory response is also involved in SCI [31]. Research indicates that early TNF‐a and IL‐1β increases in the lumbar spinal cord following SCI, and later IL‐6 increases, are inversely linked to reduced mechanical pain threshold [32]. We subsequently measured the levels of inflammation in the injured rat model and found that inflammatory markers (IL‐1β, IL‐6, and TNF‐a) were significantly upregulated. However, STVNa could significantly inhibit the expression of these factors. These results suggest STVNa can attenuate SCI by reducing inflammation and oxidative stress. The limitations of this study include the need for further investigation into the optimal duration and timing of STVNa administration, which should be explored through preclinical studies and clinical trials.

The outcome of our study revealed that STVNa could alleviate neurological function and histological changes in the spinal cord after SCI via multitarget mechanisms, such as antioxidative stress and anti‐inflammation. These findings have offered a novel perspective for the improved treatment of SCI.

## Author Contributions

T.Z. performed the experiments and data analysis. L.C. conceived and designed the study. T.Z., T.Z., and H.Y. acquired data. All authors read and approved the manuscript.

## Ethics Statement

The Care and Use Committee of Shandong University approved all experimental animal procedures. I confirm that all the research meets ethical guidelines and adheres to the legal requirements of the study country.

## Conflicts of Interest

The authors declare no conflicts of interest.

## Data Availability

All data generated or analyzed during this study are included in this article.
